# Extracranial metastases in secondary glioblastoma multiforme: a case report

**DOI:** 10.1186/s12883-020-01959-y

**Published:** 2020-10-21

**Authors:** Jessica Rossi, Lucia Giaccherini, Francesco Cavallieri, Manuela Napoli, Claudio Moratti, Elisabetta Froio, Silvia Serra, Alessandro Fraternali, Reza Ghadirpour, Salvatore Cozzi, Patrizia Ciammella, Corrado Iaccarino, Rosario Pascarella, Franco Valzania, Anna Pisanello

**Affiliations:** 1 Department of Biomedical, Metabolic, and Neural Sciences, University of Modena and Reggio Emilia, Modena, Italy; 2 Radiation Oncology Unit, Oncological Department and Advanced Technologies, AUSL-IRCCS of Reggio Emilia, Reggio Emilia, Italy; 3 Neurology Unit, Neuromotor and Rehabilitation Department, AUSL-IRCCS of Reggio Emilia, Reggio Emilia, Italy; 4 Clinical and Experimental Medicine PhD Program, University of Modena and Reggio Emilia, Modena, Italy; 5 Neuroradiology Service, Department of Diagnostic Imaging and Laboratory Medicine, AUSL-IRCCS of Reggio Emilia, Reggio Emilia, Italy; 6 Pathological Anatomy Service, Oncology Department and Advanced Technologies, AUSL-IRCCS of Reggio Emilia, Reggio Emilia, Italy; 7 Nuclear Medicine Service, Oncology Department and Advanced Technologies, AUSL-IRCCS of Reggio Emilia, Reggio Emilia, Italy; 8 Neurosurgery Unit, Neuromotor and Rehabilitation Department, AUSL-IRCCS of Reggio Emilia, Reggio Emilia, Italy

**Keywords:** Extracranial, Glioblastoma, IDH mutant, Metastases, Secondary

## Abstract

**Background:**

Glioblastoma (GBM) is known for its devastating intracranial infiltration and its unfavorable prognosis, while extracranial involvement is a very rare event, more commonly attributed to IDH wild-type (primary) GBM evolution.

**Case presentation:**

We present a case of a young woman with a World Health Organization (WHO) grade II Astrocytoma evolved to WHO grade IV IDH mutant glioblastoma, with subsequent development of lymphatic and bone metastases, despite the favorable biomolecular pattern and the stability of the primary brain lesion.

**Conclusions:**

Our case highlights that grade II Astrocytoma may evolve to a GBM and rarely lead to a secondary metastatic diffusion, which can progress quite rapidly; any symptoms referable to a possible systemic involvement should be carefully investigated.

## Background

Glioblastoma (GBM) is the most frequent and malignant brain tumor, characterized by a rapid progression and unfavorable prognosis [1]. IDH wild-type (primary) GBM develops de novo in elderly (60–80 years) patients representing approximately the 90% of all cases of GBM, while IDH mutant (secondary) GBM is typical of younger people, has a more positive biomolecular pattern and is associated with a better prognosis [1]. Despite its highly invasive nature, GBM metastases are rare and this is putatively attributed to the short overall survival and the lack of a favorable environment for an extracranial spreading of tumor cells [2, 3]. Moreover, metastases usually occur after primary GBM, while extracranial involvement from secondary GBM is extremely rare [2, 4]. Here we report the case of a patient presented with a World Health Organization (WHO) grade II astrocytoma which evolved to an IDH mutant GBM (WHO IV) with subsequent extracranial metastatic diffusion.

## Case presentation

**Fig. 1:**
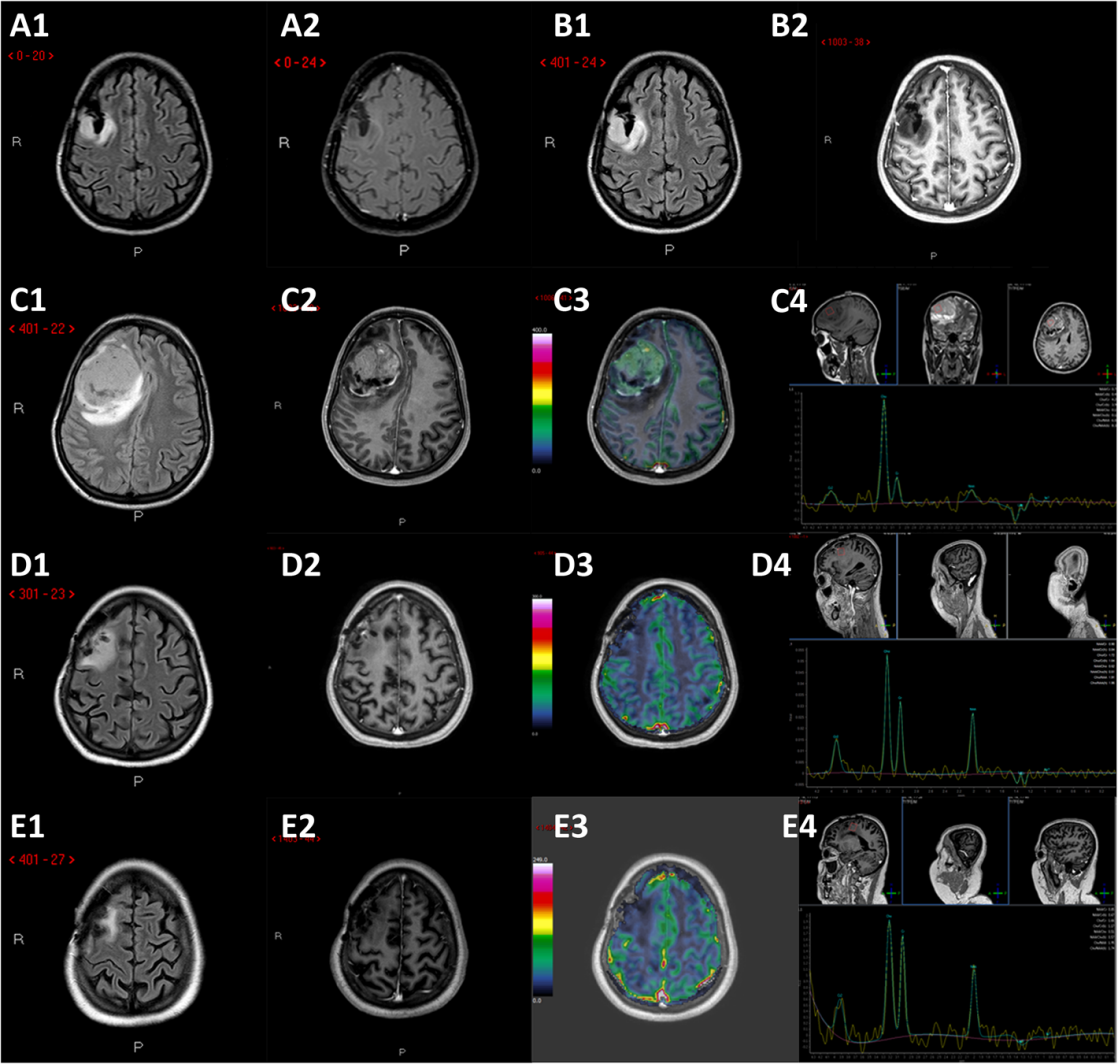
Brain-MRIs. Legend: postoperative findings after Astrocytoma tumor (WHO grade II) resection in the right frontal lobe (not shown). A, axial FLAIR (A1) and contrast-enhances T1 (A2) images 12-months FU after first surgery demonstrate small residual tumor posterior to surgical cavity without any enhancing portions. B, axial FLAIR (B1) and contrast-enhances T1 (B2) obtained after 14 months shows minimal residual tumor enlarge without enhancement. C, axial FLAIR (C1), contrast-enhances T1(C2), corresponding DSC perfusion CBV map (C3) and Single-Voxel Spettroscopy (5 months after second-surgery): progression-disease with right-frontal heterogeneously enhancing mass (C2) with surrounding FLAIR signal hyperintensity (C1), elevated cerebral blood flow (C3) and abnormally elevated Cho/NAA ratio (C4), found to be a Glioblastoma (WHO grade IV). D, axial FLAIR (D1), contrast-enhances T1(D2), perfusion CBV map (D3) and Single-Voxel Spettroscopy (D4) 6 months after third surgery: gross total resection of enhancing tumor (D2) with minimal surrounding nonenhancing white matter signal abnormality (D1) and focal dubious rCBV elevation (D3). E, axial FLAIR (E1), contrast-enhances T1(E2), perfusion CBV map (E3) and Single-Voxel Spettroscopy (E4) 12 months after third-surgery: substantial stability of the gross total resection of enhancing tumor (E2) with persistence of both minimal surrounding nonenhancing white matter signal abnormality (E1) without focal rCBV elevation (E3)

**Fig. 2:**
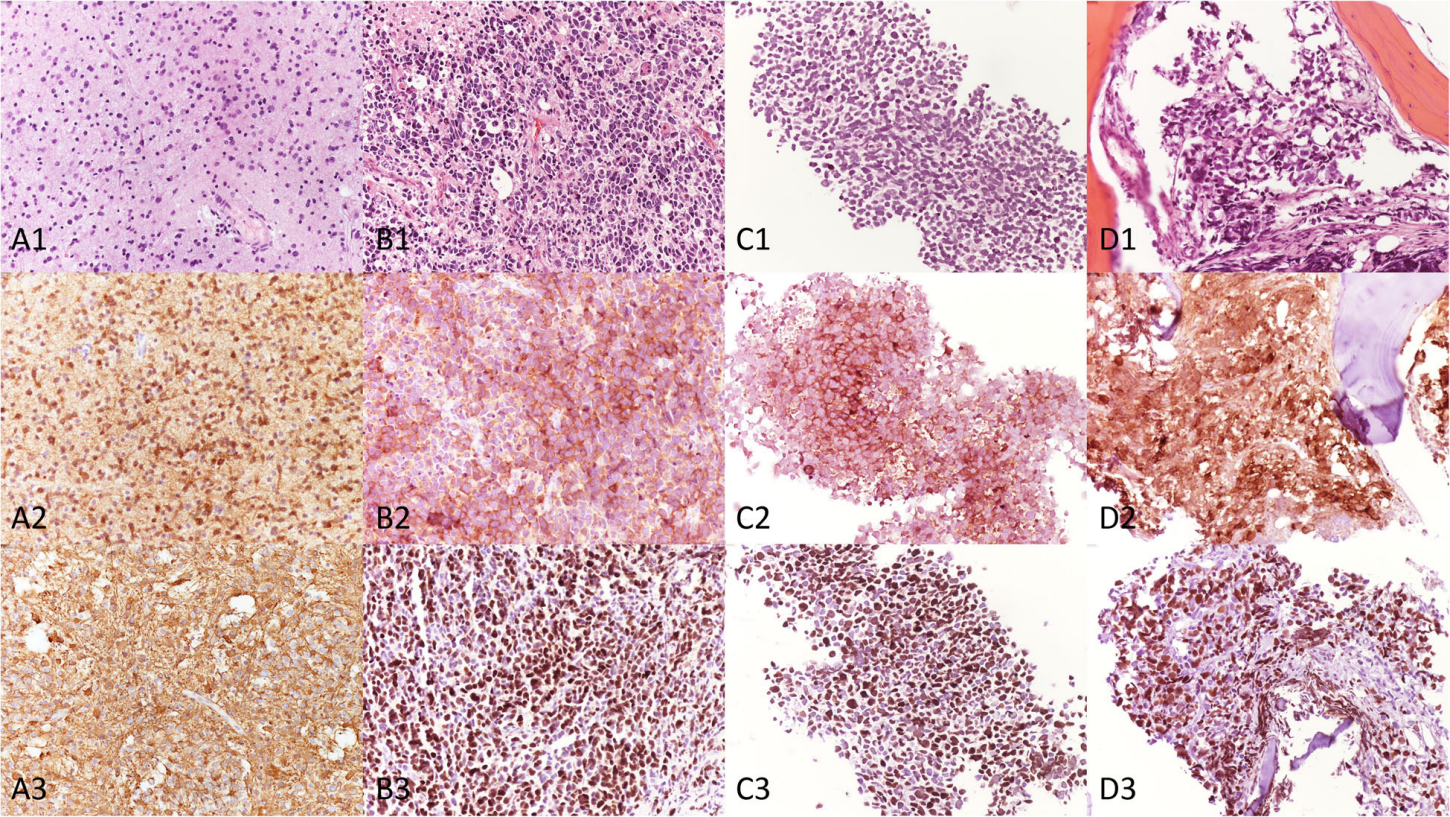
Histopathological examinations. Legend: histopathological examination of the primary brain lesion after the second and third surgery (A, B,), of the lymph node biopsy (C), and bone marrow biopsy performed at the right iliac wing (D). A, Astrocytoma shows slight to focally moderate hypercellularity composed of dark angulated nuclei without nucleoli and uneven cell distribution. Mitoses are very rare (A1, HE 20x). Neoplastic cells show cytoplasmic and nuclear staining for IDH1 (A2, 20x) and are immunoreactive for GFAP (A3, 20x). B, Glioblastoma shows hypercellularity, composed of highly pleomorphic cells with hyperchromatic nuclei, high mitotic index, endothelial proliferation and extensive areas of necrosis (B1, HE 20x). These cells are immunoreactive for IDH1 (B2, 20x) and OLIG2 (B3, 20x). C, the lymph node is complete compromised by a neoplasm composed of small and medium cells with hyperchromatic and pleomorphic nuclei and mitoses (C1, HE 20x). These cells show positivity to IDH1 (C2, 20x) and OLIG2 (C3, 20x). D, in the bone marrow neoplastic cells are similar to those observed in the primary tumor and in the lymph node (D1, HE 20x) with similar immunohistochemical stains to IDH1 (D2, 20x) and OLIG2 (D3, 20x)

**Fig. 3:**
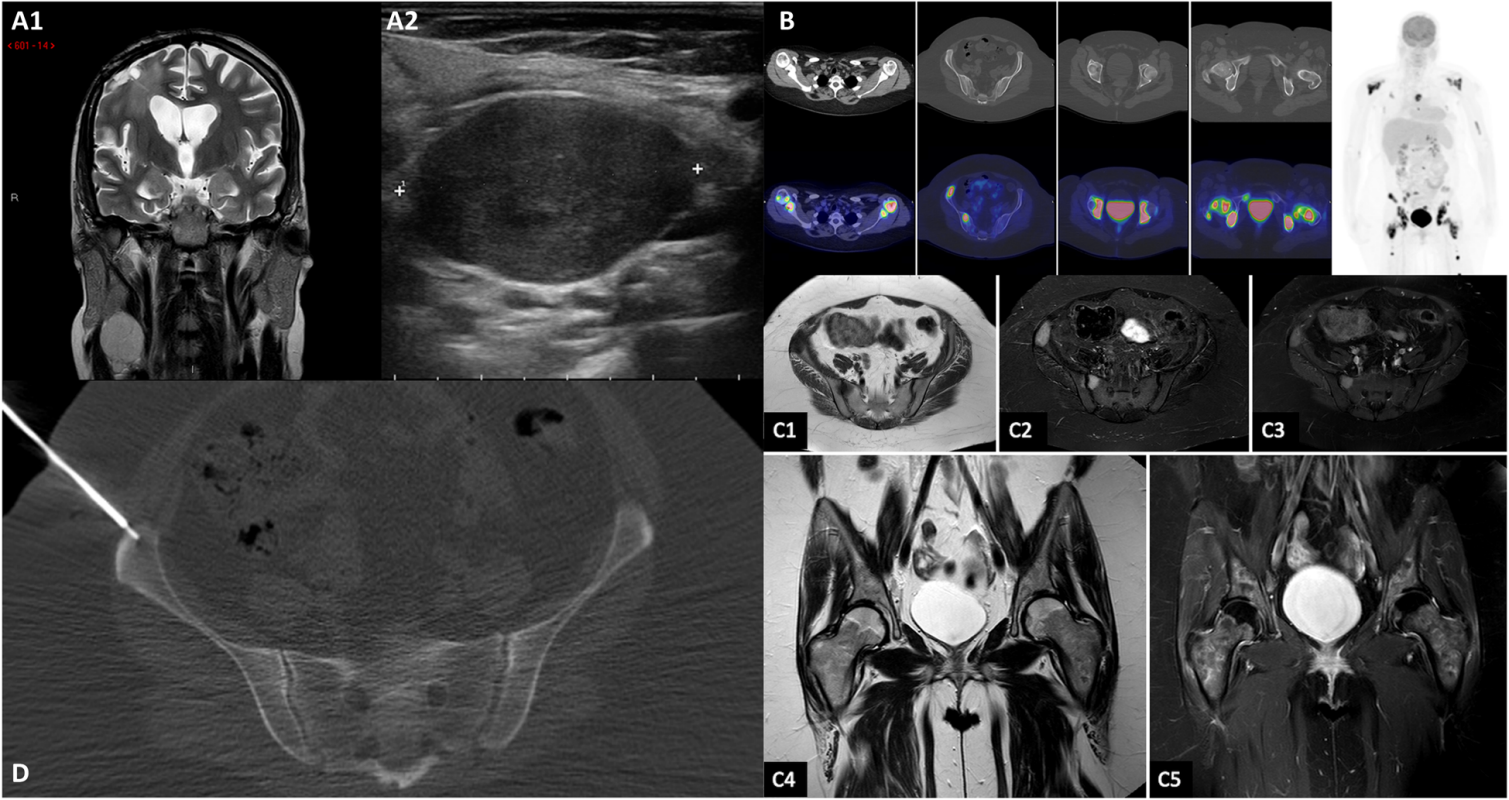
Brain and pelvic MRI, Ultrasound, Whole body 18F-FDG PET/CT scan, CT guided biopsy. Legend: Coronal T2-wighted image shows the contemporary stability of the right frontal lesion (A1) with the appearance of a right cervical mass (A1, red arrow). Ultrasound in the right lateral cervical site confirms that the lymph node is increased in size (maximum diameter of 3.5 cm) with pathological structure (A2). Whole body 18F-FDG PET/CT scan shows multiple increased 18F-FDG uptake areas (B). None of them correlates with significant osteostructural alteration on CT scan. Pelvic MRI demonstrates focal signal alterations at sacrum and iliac wing on the right side on T1-weighted axial (C1), STIR (C2), T1 SPIR with Gd (C3), and diffuse signal alteration of both hips and proximal third of the femurs on T2-weighted coronal (C4) and T1 SPIR with Gd (C5). CT guided biopsy performed at the right iliac wing, based on PET images and MRI (D)

**Fig. 4:**
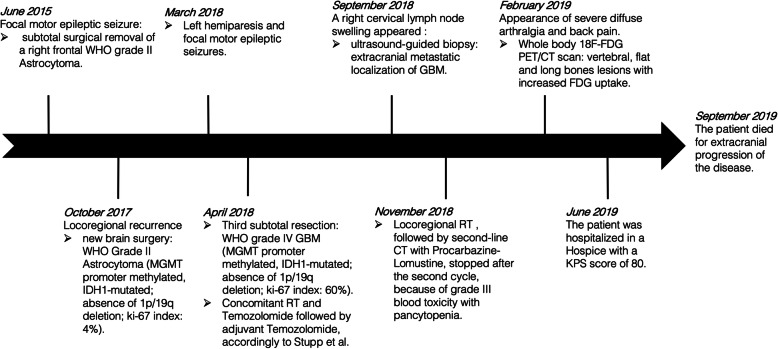
Timeline of the patient’s history. Legend: timeline of the patient’s history

A 29-year-old healthy left-handed woman, admitted to another institution after the appearance of a focal motor epileptic seizure, underwent subtotal surgical removal of a right frontal WHO grade II Astrocytoma in June 2015 (Fig. 1, A1–2). The lesion remained stable at the following six-months follow-up MRI studies until October 2017, when the patient underwent a new brain surgery for neuroradiological findings of locoregional recurrence (Fig. 1, B1–2), without any clinical worsening. Histological investigations confirmed a WHO Grade II Astrocytoma (MGMT promoter methylated, IDH1-mutated; absence of 1p/19q deletion; ki-67 index: 4%; Fig. 2, A1–3). The Karnofsky Performance Status (KPS) score at discharge was 100. Five months after the second surgery the patient presented a sudden clinical worsening, with the appearance of left hemiparesis and focal motor epileptic seizures affecting the left upper limb. A new Brain MRI documented a massive recurrence of the right frontal lesion with evident signs of grading change (Fig. 1, C1–4). This motivated the third surgery performed in April 2018, which led to a subtotal resection. Histological investigations revealed a WHO grade IV (MGMT promoter methylated, IDH1-mutated; absence of 1p/19q deletion; ki-67 index: 60%; Fig. 2, B1–3). The patient was initially treated accordingly to the protocol recommended by Stupp et al. [5] with concomitant radiation therapy (2 Gy given five days per week for six weeks, total dose: 60 Gy) and chemotherapy with Temozolomide (75 mg/m2 per day for six weeks), followed by adjuvant chemotherapy with Temozolomide (200 mg/m2 for 5/28 days). Unfortunately, the adjuvant chemotherapy with Temozolomide was early suspended after two cycles because of grade III blood toxicity with pancytopenia. In September 2018, a right cervical lymph node swelling appeared. An ultrasound of the neck showed some lymph nodes increased in size (maximum diameter of 3.5 cm) with pathological structure in the right lateral cervical site (Fig. 3, A1–2). A needle biopsy was performed and, according to the immunohistochemical, biomolecular and histological results, confirmed the presence of an extracranial metastatic localization of GBM (Fig. 2, C1–3). A thoracic and abdominal CT scan ruled out further diffusion to other sites, and a brain MRI didn’t show any sign of progression of the primary lesion. Locoregional radiotherapy (6 Gy given for five consecutive fractions, total dose: 30 Gy), was performed leading to a complete remission of the 18F-FDG uptake in the right lateral cervical region at the five months follow-up. In addition, a second-line chemotherapy with Procarbazine-Lomustine was started, but it was stopped after the second cycle, because of grade III blood toxicity with pancytopenia. About two months after the last radiotherapy treatment, the patient complained of the appearance of severe diffuse drug resistant arthralgia and back pain, without any worsening at neurologic examination. A whole body 18F-FDG PET/CT scan showed multiple increased 18F-FDG uptake areas involving ilium bilaterally and the proximal third of the femurs (SUV max = 25), scapula and humeral head bilaterally (SUV max = 22), sternum (SUV max = 20), some ribs, some vertebrae and the sacrum (SUV max = 15; Fig. 3, B). None of the 18F-FDG uptake areas correlated with significant structural alteration on CT scan. Pelvis MRI demonstrated signal alteration areas compatible with GBM metastatic bone infiltration (Fig. 3, C1–5). Based on PET images and MRI, a CT guided biopsy was performed at the right iliac wing (Fig. 3, D) and the histopathological examination confirmed the presence of secondary bone localizations of GBM (Fig. 2, D1–3). In the face of this diffusion, brain-MRI documented stability of the brain disease for 14 months (Fig. 1, D1–4, E1–4; Fig. 3, A1) and no clinical or radiological signs of progression were discovered in the irradiated lymph node site for approximately 1-year. About 12 months after the appearance of the first metastatic site, the patient was hospitalized in a Hospice with a KPS score of 70 where died for extracranial progression of the disease and subsequent sepsis at the age of 33-years. Figure 4 summarizes the timeline of the patient’s history.

## Discussion and conclusion

The vast majority of glioblastomas (90%) develop rapidly de novo in elderly patients and are called IDH wild-type (primary) GBM [1]. IDH mutant (secondary) glioblastomas are rarer and progress from low-grade diffuse astrocytoma or anaplastic astrocytoma [6]. They manifest in younger patients, are preferentially located in the frontal lobe and seizures are the initial symptom in approximately 70% of patients [1]. They develop through distinct genetic and biological pathways, showing IDH1 mutations and a hypermethylation phenotype (which are absent in primary glioblastomas) and carry a significantly better prognosis [6].

Extracranial GBM metastases are extremely rare, affecting 0.4–0.5% of all patients with GBM [2]. The rarity of this phenomenon is attributed to: 1) the rapid intracranial progression of the GBM, leading to low overall survival (OS) and leaving not sufficient time for successful systemic diffusion; 2) the lack of favorable cerebral environment for an extracranial tumor cells spreading (e.g., the dura mater, the thickened basement membrane, and the blood-brain barrier [7]); 3) the absence of a suitable environment for multiplication of metastatic cells, which seem to have preferential adhesion to the neural stroma [3]. Younger and otherwise healthier patients are more susceptible to develop extracranial metastases, most probably due to a longer OS compared to elderly GBM patients with multiple chronic illnesses [7]. The bone, lymph nodes, and lung are among the most commonly affected sites [8], but also liver, soft tissue, and the skin can be involved [7]. Among the lymph node metastases, 62% are situated in the cervical areas, often ipsilateral to the site of craniotomy but sometimes bilateral [9]. The mean time between the diagnosis of metastases and death is about 12 months [2], with a better prognosis of GBM metastatic to the neck and a worse prognosis for GBM metastatic to the lung and to the liver [3]. Risk factors and pathogenetic mechanisms of GBM metastasis are not yet well understood, but it’s hypothesized that GBM cells invasion through the vein system or directly through the dura and the breakdown of the blood–brain barrier could favor GBM cell diffusion through the systemic circulation [2]. Moreover, recent studies have demonstrated the existence of lymphatic vessels in the meninges [10], which can play an important role in GBM dissemination. This network of lymph vessels is called “glymphatic system” and likely drain into deep cervical lymph nodes [10], giving a possible explanation for the relatively high occurrence of cervical nodal metastases, sometimes in the absence of recurrence in the surgical scar or even without any pre-existing surgical procedure [11]. Despite surgery on the primary lesion have been reported in association with hematogenous seeding of tumor cells [7], around 10% of the reported cases of extracranial GBM diffusion occurred without surgical intervention [3], and recent studies did not find evidence for a tumoral cells release induced by surgery [12]. If metastases from primary glioblastomas are rare, extracranial dissemination from a secondary GBM is an exception [9, 13]. We performed a research in PubMed, looking for other cases of secondary GBM with extracranial metastasis, and we found seven other cases [4, 9, 13–17], including one case of peritoneal dissemination following ventricle-peritoneal shunt (Table 1) [13]. Our patient is the eighth described case of this very rare occurrence, as she presented systemic metastases from secondary glioblastoma despite a prognostically favorable biomolecular pattern. Furthermore, the progressive systemic involvement occurred without any sign of progression of the intracranial pathology, countering the hypothesis that local tumor progression is a major cause of systemic metastases [18, 19]. Moreover, it’s known that GBM spreading plays a minor role for the clinical course and prognosis of affected patients, but this phenomenon is putatively more common than assumed as systemic metastases of the GBM are found in 6–25% of autopsies of affected patients [7] and tumoral circulant cells has been detected in 20% of GBM cases [13]. We can suppose that a longer disease progression due to an initially good histological and molecular pattern may increase the likelihood of a systemic involvement from GBM. This would be in agreement with the hypothesis that the occurrence of metastases from low-grade gliomas may happen before these tumors undergo grade increase, enabling the ability of tumor cells to settle in metastatic sites [3]. So far, the treatment of extra-CNS metastasis varies widely and there was no substantial treatment progress over the recent decades [20, 21]. In our patient, the locoregional radiotherapy allowed good control of lymph node disease even at a distance of about 8–10 months after treatment. Regarding bone metastases, it is described that the spine (73%) is the most common site of involvement, followed by the ribs (23%), sternum (18%), skull (14%), and acetabulum (9%) [9].

**Table 1:** Summary of case reports about secondary GBM with extracranial metastasis

Authors and Year	Age (years)	Gender	Baseline histology	Primary lesion biomolecular pattern	Primary lesion treatment	Time between primary lesion and GBM (months)	Secondary GBM biomolecular pattern	Secondary GBM treatment	Time between secondary GBM and metastases (months)	Metastases localization	Metastases treatment	Survival time from metastases (months)
Cervio et al., 2001 [4]	44	Male	WHO grade II Oligodendroastrocytoma	Positivity for GFAP, Ki-67 index: 4%	Surgery followed by CT (4 cicles of Lomustine)	141	Positivity for EGFR; negativity for P53; absence of 1p/19q deletion; ki-67 index: 22%	Surgery, followed by WBRT	12	Bone (pelvis, femurs, sternum, shoulders, ribs, dorsal and lumbar vertebrae)	CT (Etoposide 50 mg per day)	5
Ueda et al., 2004 [14]	42	Male	WHO grade II Astrocytoma	Positivity for GFAP, DNA-PKcs, Ku70, Ku86, MIB-1	Two surgeries and RT for recurrence	73	Positivity for GFAP, vimentin, IGFBP2, BAD; negativity for p53, mdm2, p16; weak positivity for Ku70; MIB-1 positive rate: 21% at the third surgery, 28% at the fourth surgery	Surgery, followed by CT (3 cycles of ranimustine) and a fourth surgery	0	Cervical spinal cord, both lungs, epicardium, right kidney, pancreas, liver, left cervical and auricle soft tissue, bones (left clavicle, left ribs, cervical and thoracic vertebrae), and multiple lymph nodes	CT (3 cycles of ranimustina-9, followed by surgery (on intracranial and subcutaneous lesion)	18
Zhen et al., 2010 [9]	25	Male	WHO grade II Astrocytoma	Weak positivity for p53 and EGFR	Surgery	13	Positivity for p53, EGFR, MGMT, syn, CD56, Ki-67; negativity for CD3, CD99, GFAP, S-100, VEGF, Vimentin, Leu-7, Olig-2, Nestin, Neu-N	Surgery, followed by RT	2	Right cervical lymph node; bone (mainly pelvic bone)	Neck dissection, followed by CT	Not reported
Blume et al., 2013 [15]	35	Male	WHO grade II Astrocytoma	Positivity for GFAP, MAP 2c, WT1, IDH1- mutated. Nuclear accumulation of p53 in a subpopulation of ca. 1–2%	Stereotactic biopsy and follow-up	24	Positivity for GFAP in 65% and p53 in 5–10% of tumor cells	Surgery, followed by RT, and concomitant CT with Temozolomide	36	Lung, pulmonary lymph nodes, vertebrae, cervical muscles and epidural space	Surgery on the cervical spine, followed by combined RT and CT with temozolomide	10
Taskapılıoglu et al., 2013 [16]	30	Female	WHO grade III Anaplastic Oligodendroglioma	Positivity for S-100 and focal GFAP expression	Surgery	7	Positivity for GFAP and p53	Surgery, followed by RT and CT (7 cycles of Temozolomide)	10	Right parotid gland; right cervical, preauricular and retro auricular lymph nodes; bone (left ischium)	parotidectomy and radical neck dissection	6
Granados et al., 2018 [13]	15	Female	Low grade Astrocytoma	Not described	Ventriculoperitoneal shunt, followed by RT; subsequent stereotactic radiosurgery for a relapse	49	Not described	CT with Temozolomide	0	Posterior wall of the uterus, lateral wall of the rectum, II hepatic segment, right kidney, and peritoneal layers	Palliative care	1
Rodrigues et al., 2020 [17]	32	Male	WHO grade III Anaplastic Astrocytoma	Positivity for GFAP, mutated IDH1 (R132H) negative, Ki-67 index: 8%	Stereotactic biopsy, followed by CT with Temozolomide and RT	19	Positivity for GFAP, Vimentin, S100β, SOX-2, Nestin	RT, CT and palliative surgery	0	Cervical nodes, neck, ribs, thoracic spine and the scapula	RT, CT and two palliative surgeries	3
Our case	29	Female	WHO grade II Astrocytoma	MGMT promoter methylated, IDH1-mutated; absence of 1p/19q deletion; ki-67 index: 4%	Two surgeries	34	MGMT promoter methylated, IDH1-mutated; absence of 1p/19q deletion; ki-67 index: 60%	Surgery, followed by RT, concomitant and adjuvant CT with Temozolomide	5	Right cervical lymph node; bone (ilium, femurs, scapula, humeral head bilaterally, sternum, ribs, vertebrae and the sacrum)	Locoregional RT and CT	9

*Abbreviations*: *BAD* BCL2 antagonist of cell death, *CT* Chemotherapy, *EGFR* Epidermal Growth Factor Receptor, *GBM* Glioblastoma, *GFAP* Glial Fibrillary Acidic Protein, *GFBP-2* Insulin-like growth factor-binding protein 2, *IDH1* Isocitrate Dehydrogenase 1, *MGMT* O-6-methylguanine-DNA methyltransferase, *RT* Radiation Therapy, *VEGF* Vascular Endothelial Growth Factor, *WBRT* Whole Brain Radiation Therapy

In our case, we noticed a more frequent localization of bone metastatic lesions at the joint extremities of the shoulders and hip, with bilateral and symmetrical distribution. Furthermore, in the future it could be very interesting to better investigate the “biomolecular” profile of GBM metastases, in order to assess whether they differ from the primary tumor. Indeed, metastases have been associated with specific molecular changes such as EGFR gene amplification [22] and it should be interesting to investigate their genetic features, given the rarity of this phenomenon. However, the small number of patients could be a limit to design any prospective studies. Unfortunately, in our case we were not able to investigate further the biomolecular profile including specific molecular changes such as EGFR gene amplification. This represented a limitation in our case description. Despite a favorable biomolecular pattern, Grade II Astrocytoma may shift to a GBM and lead to secondary metastatic diffusion, but it remains a very rare evolution of such tumors. With prolonged survival of young patients with GBM, the possibility of GBM cells spreading to the bloodstream may increase [3]. Bone, lymph nodes, and lung are among the most commonly affected sites and any symptoms referable to these areas should be carefully evaluated. In our case the bone metastatic lesions did not cause any appreciable alteration on CT scan. Therefore, nonspecific signs and symptoms, like local tumefactions or bone and joint pain, should never be underestimated, as they may be suggestive for a systemic diffusion of the GBM. In this context, it is always advisable to proceed to a systemic investigation of nuclear medicine and MRI, even if CT scan is negative, as an early diagnosis can help to expedite alleviation of patients’ discomfort, in an already aggressive disease process [8]. Moreover, in our patient locoregional radiotherapy has allowed good control of the lymph node disease even at a distance of about 8–10 months after treatment.

## Data Availability

Data sharing is not applicable to this article as no datasets were generated or analyzed during the current study.

## Abbreviations

CNS: Central nervous system
CT: Computed tomography
FDG: Fluorodeoxyglucose
GBM: Glioblastoma
IDH: Isocitrate dehydrogenase
MRI: Magnetic resonance imaging
MGMT: O6-methylguanine-DNA methyl-transferase
KPS: Karnofsky Performance Status
OS: Overall survival
PET: Positron Emission Tomography
SUV: Standardized uptake value
WHO: World Health Organization
